# Comparison of Dysferlin Expression in Human Skeletal Muscle with That in Monocytes for the Diagnosis of Dysferlin Myopathy

**DOI:** 10.1371/journal.pone.0029061

**Published:** 2011-12-16

**Authors:** Eduard Gallardo, Noemi de Luna, Jordi Diaz-Manera, Ricardo Rojas-García, Lidia Gonzalez-Quereda, Bàrbara Flix, Antoine de Morrée, Silvère van der Maarel, Isabel Illa

**Affiliations:** 1 Servei de Neurologia, Laboratori de Malalties Neuromusculars, Hospital de la Santa Creu i Sant Pau i Institut de Recerca de HSCSP, Universitat Autònoma de Barcelona and Centro de Investigación Biomédica en Red de Enfermedades Neurodegenerativas (CIBERNED), Barcelona, Spain; 2 Servei de Genètica, Hospital de la Santa Creu i Sant Pau i Institut de Recerca de HSCSP, Universitat Autònoma and Centro de Investigación en Red de Enfermedades Raras (CIBERER), Barcelona, Spain; 3 Department of Human Genetics, Leiden University Medical Center, Leiden, The Netherlands; Charité Universitätsmedizin Berlin, NeuroCure Clinical Research Center, Germany

## Abstract

**Background:**

Dysferlinopathies are caused by mutations in the dysferlin gene (*DYSF*). Diagnosis is complex due to the high clinical variability of the disease and because dysferlin expression in the muscle biopsy may be secondarily reduced due to a primary defect in some other gene. Dysferlin is also expressed in peripheral blood monocytes (PBM). Studying dysferlin in monocytes is used for the diagnosis of dysferlin myopathies. The aim of the study was to determine whether dysferlin expression in PBM correlates with that in skeletal muscle.

**Methodology/Principal Findings:**

Using western-blot (WB) we quantified dysferlin expression in PBM from 21 pathological controls with other myopathies in whom mutations in *DYSF* were excluded and from 17 patients who had dysferlinopathy and two mutations in *DYSF*. Results were compared with protein expression in muscle by WB and immunohistochemistry (IH). We found a good correlation between skeletal muscle and monocytes using WB. However, IH results were misleading because abnormal expression of dysferlin was also observed in 13/21 pathological controls.

**Conclusions/Significance:**

The analysis of dysferlin protein expression in PBM is helpful when: 1) the skeletal muscle IH pattern is abnormal or 2) when muscle WB can not be performed either because muscle sample is lacking or insufficient or because the muscle biopsy is taken from a muscle at an end-stage and it mainly consists of fat and fibrotic tissue.

## Introduction

Dysferlinopathy is a type of muscular dystrophy characterized by mutations in the *DYSF* gene [1], [2]. It has a wide phenotypic variability that includes distal forms, such as Miyoshi myopathy (MM) and myopathy with onset in the distal anterior compartment of the legs (DACM), a proximal form known as limb girdle muscular dystrophy 2B (LGMD2B), and hyperCKemia [3], [4], [5], [6], [7], [8], [9]. A congenital form and an early-onset form have also been described [10]. In spite of the clinical variability, it has been observed that muscle MRI shows that both distal and proximal muscles are impaired from the onset of the disease in all phenotypes [11]. As well as in skeletal muscle, dysferlin is expressed in peripheral blood CD14^+^ monocytes (PBM), as reported in a series of 12 patients with MM or LGMD2B [12]. The findings from this latter study suggested that studying the expression of dysferlin protein in these more accessible cells could be a reliable method to diagnose dysferlinopathies and a valid alternative to muscle biopsy. However, the expression of dysferlin in monocytes was not compared with that in skeletal muscle. Wein *et al* performed a flow cytometry study in a series of 6 patients with dysferlin myopathy and found that dysferlin expression in PBM differed between patients and controls, although the protocol would not allow quantification of dysferlin using the antibody NCL-Hamlet [13]. Furthermore, the authors did not compare the expression of dysferlin between blood and muscle. The present study was performed to assess whether there is a correlation between the expression of dysferlin in skeletal muscle and that in PBM. We analysed a large series of genetically characterized patients comparing the expression of dysferlin in skeletal muscle by immunohistochemistry (IH) and western blot (WB) with that in PBM.

## Results

### Dysferlin protein is expressed as a double band in PBM

**Figure 1:**
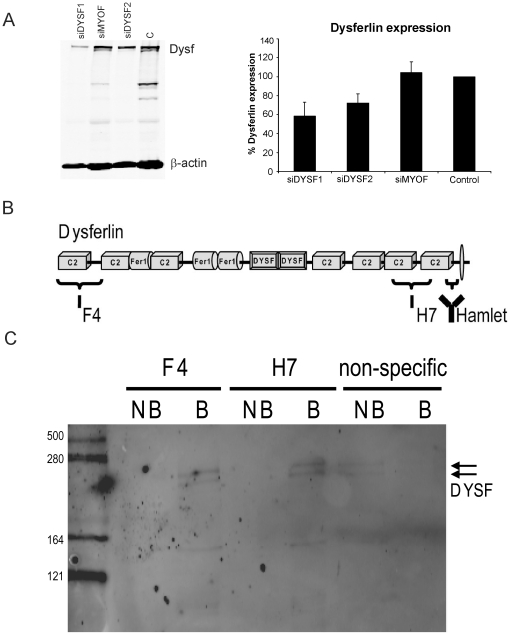
Characterization of the double band corresponding to dysferlin observed in CD14+ PBM by WB. A) Dysferlin siRNA efficiently reduced both dysferlin bands in PBM. Representative WB of monocytes from a healthy donor treated with siRNA showed that siDYSF efficiently reduced the two bands corresponding to dysferlin. On the right, quantification of WB from silenced monocyte samples, corresponding to four independent experiments, showed that when dysferlin was knocked down, levels diminished from 100% in Control (C) and 104.2±11.3% in siMYOF to 58.4±14.8% with siDYSF1 (p<0.05) and 72.2±9.8% with siDYSF2 (p<0.05), error bars indicate standard desviation. B) Schematic overview of the dysferlin protein and the epitopes recognized by F4, H7 and Hamlet antibodies. The three affinity binders together cover the complete dysferlin protein sequence. C) Both F4 and H7 antibodies can immunoprecipitate the dysferlin doublet from PBM. Bound (B) and non-bound (NB) fractions were analyzed by WB for dysferlin. The arrow denotes the dysferlin doublet observed in the bound fraction using F4 and H7 and in the non-bound fraction using an antibody against β-amyloid.

WB analysis showed dysferlin was expressed as a double band in PBM. The molecular weight of the upper band corresponded to the single band expressed in skeletal muscle (234 kDa) and the lower band was approximately 8–10 kDa smaller. When monocytes were transfected with dysferlin siRNAs, the intensity of the two bands decreased significantly. Control siRNAs (siMYOF and non-target siRNA) did not affect the protein levels of the doublet (Figure 1A). In immunoprecipitation studies using both F4 and H7 antibodies (Figure 1B) the doublet from PBM was pulled down (Figure 1C). When a control antibody against β-amyloid was used we observed the dysferlin doublet only in the non-bound fraction (Figure 1C). These results indicate that both bands correspond to dysferlin protein and support using both of them to quantify dysferlin in PBM. In addition, in all patients with two mutations in *DYSF* in whom the monocytes test was performed both bands were absent.

### Mutation analysis of *DYSF*

We found two mutations in all 17 patients (Table 1). In order to rule out the presence of two mutations in the same allele of *DYSF*, parents of patients P_1, P_2, P_4, P_14, P_15 P_16, P_17 (table 1) were analyzed and one of the familial mutations was found in each one. In P_6, P_9, P_11 and P_12 (Table 1) only one of the two mutations was found in their sons/daughters. In the remaining 6 patients (P_3, P_5, P_7, P_8, P_10 and P_13) (Table 1), DNA/RNA sample from relatives was not available [3].

**Table 1:** Summary of dysferlin myopathy patients: Correlation with protein expression in PBM, skeletal muscle and mutations.

Patient	Sex	Age	Phenotype	PBM WB (%)	Muscle WB (%)	Muscle IH	Nucleotide change DYSF gene	Protein change
P_1	M	31	MM	0	0	Sarcoplasm granular pattern	c.5194G>Tc.5999G>A	p.Glu1732Xp.Arg2000Gln
P_2	M		MM	0	0	Absent	c.1555G>Ac.1555G>A	p.Gly519Argp.Gly519Arg
P_3	M	37	MM	0	0	Absent	c.5159delGc.5159delG	p.Arg1720LeufsX2p.Arg1720LeufsX2
P_4	M	26	LGMD2B	0	0	Absent	c.5509G>Ac.5903G>A	p.Asp1837Asnp.Trp1968X
P_5	F	27	LGMD2B	0	0.2	Absent	c.5979dupAc.5979dupA	p.Glu1994ArgfsX3p.Glu1994ArgfsX3
P_6	F	38	LGMD2B	0	0	Absent	c.154T>Cc.701G>A	p.Trp52Argp.Gly234Glu
P_7	M	30	LGMD2B	0	0	Sarcoplasm granular pattern	c.2779delGc. 4410+13T>G	p.Ala927LeufsX21
P_8	M	36	LGMD2B	0.9	1.5	Patchy sarcolemmal pattern	c.2055+1G>Ac.3805G>T	p.Glu1269X
P_9	F	49	LGMD2B	0	0	Absent	c.4354C>Ac.4354C>A	p.Pro1452Thrp.Pro1452Thr
P_10	F	63	LGMD2B	0	0	ABSENT	c.5525+3A>Gc.5525+3A>G	
P_11	F	44	LGMD2B	0	0	Absent	c.5159delGc.5979dupA	p.Arg1720LeufsX2p.Glu1994ArgfsX3
P_12	F	73	LGMD2B	0	0	Absent	c.2875C>Tc.2875C>T	p.Arg959Trpp.Arg959Trp
P_13	M		MM	0	0	Patchy sarcolemmal pattern	c.4003G>Ac.4003G>A	p.Glu1335Lysp.Glu1335Lys
P_14	F	28	DACM	0	0	Absent	c.5594delGc.5594delG	p.Gly1865AlafsX 101p.Gly1865AlafsX 101
P_15	F	29	MM	0	18	Patchy sarcolemmal pattern	c.5979dupAc.9124C>T	p.Glu1994ArgfsX3p.Arg2042Cys
P_16	M	17	LGMD2B	0	0	Sarcoplasm granular pattern	c.4882G>Ac.4882G>A	p.Gly1628Argp.Gly1628Arg
P_17	M	5	LGMD2B	0.2	0	Absent	c.2779delGc.2779delG	p.Ala927LeufsX21p.Ala927LeufsX21

M- male; F- female; MM: Miyoshi myopathy; LGMD2B: limb girdle muscular dystrophy; DACM: Distal anterior compartment myopathy.

The mutations of patients P_1, P_2, P_4, P_5, P_6, P_10, P_13, P_14 [12], [17], P_15 [8] and P_17 [26] have been previously described. The intronic mutation, c. 4410+13T>G, of P_7 has been previously described in the Leyden Muscular Dystrophy web page (www.dmd.nl) (Accession number used NM_003494.2).

### The expression of dysferlin in monocytes correlated with that in skeletal muscle

**Figure 2:**
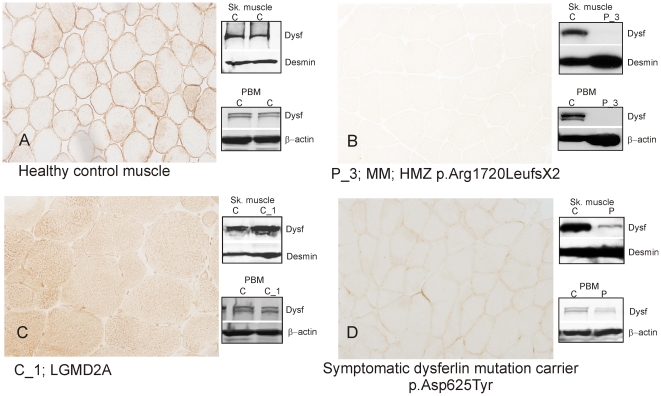
Correlation between dysferlin expression in skeletal muscle, detected by IH and WB, and WB of PBM. A) Healthy control with normal expression of dysferlin in PBM: Dysferlin is expressed in the sarcolemma of muscle fibres. WB of skeletal muscle and PBM showed normal levels of dysferlin. B) Dysferlinopathy patient (P_3): Dysferlin is absent in sarcolemma by IH, WB and in PBM. C) Patient with LGMD2A (C_1) showing abnormal expression of dysferlin in skeletal muscle by IH but normal levels in skeletal muscle and PBM WB. D) Symptomatic carrier of one mutation in *DYSF*: muscle IH and WB show reduced expression that was confirmed in PBM. HMZ: homozygous.

In 21 cases with normal expression of dysferlin in PBM (114.3±17.8%), skeletal muscle WB was also normal (105±16.3%) (Figure 2A and Table 2).

**Table 2:** Summary of patients with normal expression of dysferlin included in this study.

Patient	Sex	Age	Phentoype	PBMWB(%)	MuscleWB(%)	MuscleIH	Gene	Nucleotide change	Protein change
C_1	M	19	LGMD2A	121.6	110.5	2	*CAPN3*	c.2362_2363delinsTCATCTc.2362_2363delinsTCATCT	p.Arg788SerfsX14p.Arg788SerfsX14
C_2	M	48	BMD	100	96	3	*DYS*	c.1704+2T>A	p.Cys569ValfsX18
C_3	M	73	POMPE	116.2	93.6	3	GAA	c.1195_1G>Ac.1856G>A	IVS17p.Ser619Asn
C_4	F	40	POMPE	125	118.7	3	*GAA*	c.32_13T>Gc.236_246delCCACACAGTGC	IVS1-13 T>Gp.Pro79ArgfsX13
C_5	F	56	POMPE	110.5	100	N	*GAA*	c.32_13T>Gc.2600_2604delTGCTGinsA	IVS1-13 T>G; p.Val1867GlufsX19
C_6	F	30	ANO5	100	100	N	*ANO5*	c.191dupAc.1627dupA	p.Asn64LysfsX15p.Met543AsnfsX11
C_7	M	35	Mitochondrial myopathy	100	98.6	N			
C_8	F	46	Nonaka	110.5	100	1	*GNE*	c.934G>Ac.1519A>C	p.Gly312Argp.Thr507Pro
C_9*	M	62	HyperCKs	140	105.2	1			
C_10	F		McArdle	100	100	N	*PYGM*	c.1979C>Tc.1760T>C	p.Ala660Aspp.Leu587Pro
C_11	F	59	VCP	149.4	130.1	N	*VCP*	c.277C>T	p.Arg93Cys
C_12	M	55	PM	100	77.5	N			
C_13	F	57	PM	98.3	89.9	3			
C_14*	M	43	LGMD	100	99.5	3			
C_15*	M	46	LGMD	150	132.6	3			
C_16*	M	43	LGMD	100	100	2			
C_17*	F	29	HyperCKs	98.3	93.3	N			
C_18*	M	61	Myalgias hiperCKs	140	151.1	2			
C_19*	M	59	Muscular dystrophy	114.9	108	1			
C_20*	M	49	Calf atrophy	125.5	100	N			
C_21*	M	42	MM like	100	100	1			

N-Normal; 1- reduced sarcolemma; 2- Reduced sarcolemma and increased sarcoplasm staining; 3- Variable sarcoplasm staining.

*Patients with *DYSF* gene analyzed and no mutations found.

M:male; F: female; LGMD: limb girdle muscle dystrophy; VCP: valosin containing protein; PM: polimyositis; MM: Miyoshi myopathy. HMZ: homozygous.

The mutation of pathological control C_X have been previously described [38].

We performed skeletal muscle WB in 17 dysferlinopathy patients and we found that dysferlin expression was 1.1±4.3%. Similar dysferlin levels of expression were found in PBM (0.1±0.2%) (Figure 2B and Table 1). A WB of skeletal muscle and PBMs from 2 patients and 2 controls all loaded in the same gel was performed to confirm that primary deficiencies of dysferlin can be easily detected in PBMs (Figure S1).

Patients with abnormal dysferlin staining by IH showed normal levels of the protein by WB both in skeletal muscle and PBM (Figure 2C)(C_1 in Table 2).

We measured the expression of dysferlin in PBM by WB in the two previously reported symptomatic carriers [3]. The results were 37.9±3% in monocytes and 36.3±12.2% in muscle (Figure 2D).

**Figure 3:**
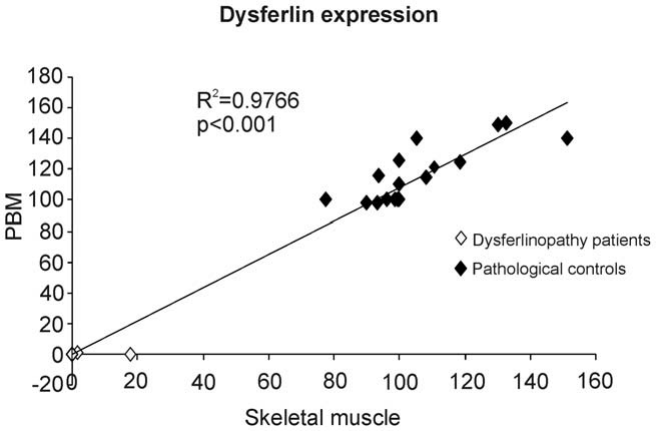
Parallelism between dysferlin expression in PBM and in skeletal muscle. Linear association between dysferlin expression in skeletal muscle (X axis) and in PBM (Y axis) were observed (R^2^ = 0.9766, p<0.001). Black diamonds show dysferlin expression in pathological controls and white diamonds show dysferlin expression in dysferlinopathy patients.

An X-Y plot was performed to confirm the parallelism between dysferlin expression in PBM and in skeletal muscle (Figure 3).

**Figure 4:**
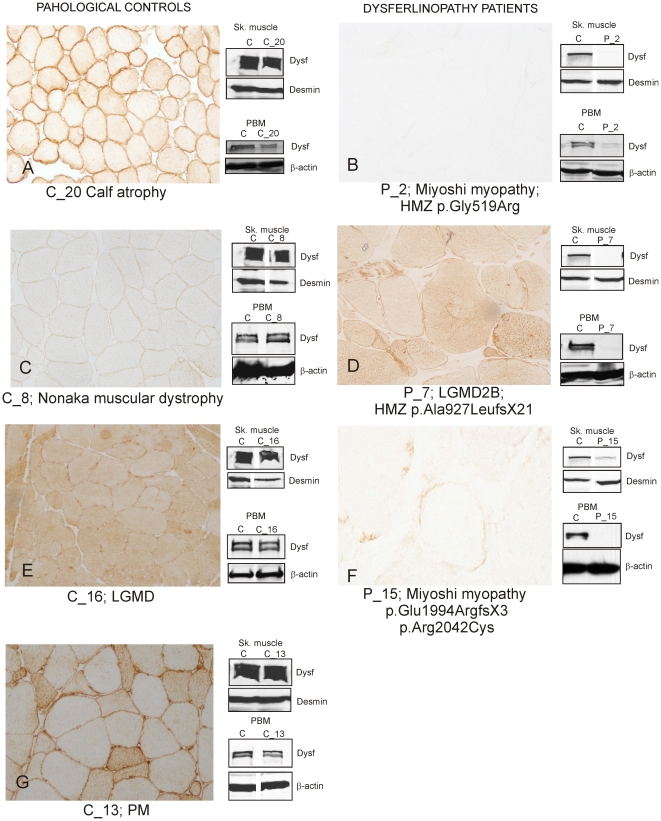
Dysferlin staining patterns in muscle biopsy from dysferlinopathy patients and from other muscle diseases. Muscle biopsies from dysferlinopathy patients show three staining patterns: A) Normal dysferlin staining in a pathological control muscle (C_20 with calf atrophy in Table 2). B) Total absence of dysferlin expression at sarcolemma and sarcoplasm (P_2 with Miyoshi myopathy in Table 1). C) reduced sarcolemmal staining in all muscles (C_8 with Nonaka myopathy in Table 2). D) A sarcoplasm granular pattern in scattered fibers was observed in some dysferlinopathy patients (P_7 with LGMD2B) in Table 1) E) Increased cytoplasmic staining of dysferlin in all muscle fibres (C_16 with LGMD in Table 2). F) A patchy staining of dysferlin was observed in the sarcolemma in some muscle fibres (P_15 with Miyoshi myopathy in Table 1). G) Some muscle biopsies had increased dysferlin staining in the cytoplasm of some fibers (P_13 with PM in Table 2).

We found absence of dysferlin staining in 11/17 patients with abnormal dysferlin expression in PBM (Figure 4B). The remaining 6 patients had some degree of dysferlin expression in muscle. Two patterns were identified: i) a sarcoplasm granular pattern in scattered fibres in 3/6 (P_1, P_7, P_16) (Figure 4D); and ii) markedly reduced but unequivocal patchy sarcolemmal expression in 3/6 (P_8, P_13 and P15) (Figure 4F). Analysis of the PBM WB in these 6 patients showed 0.1±0.3% of dysferlin expression compared to 0±0% in the 11 patients with absent dysferlin immunostaining. We did not see any association with the type of mutation or its location within *DYSF* either in these 6 patients with residual amounts of dysferlin in the muscle biopsy or the 11 patients with absence of dysferlin (Table 1). In 21 muscle biopsies from patients with normal dysferlin expression in PBM we found different dysferlin staining patterns, similar to those previously described [14]: Dysferlin expression was normal in the sarcolemma in eight patients (C_5, C_6, C_7, C_10, C_11, C_12, C_17, C_20) (Figure 4A). Of the other 13, four showed a general reduction of a continuous staining (not patchy) of the sarcolemma (C_8, C_9, C_19, C_21) (Figure 4C), three had reduced staining in the sarcolemma but increased in the cytoplasm of all muscle fibers (C_1 C_16, C_18) (Figure 4G), and six had increased signal in the cytoplasm of only some fibres with normal expression in the sarcolemma (P_2, P_3, P_4, P_13, P_14, P_15 (Figure 4H and Table 2).

## Discussion

Our study showed a reproducible relationship between dysferlin expression in skeletal muscle and in PBM by WB analysis. Analysis of 17 dysferlinopathy patients with two pathogenic mutations in the gene *DYSF* and 21 patients with other neuromuscular diseases confirmed normal levels of dysferlin in all the pathological controls and abnormal dysferlin (severe reduction or absence) in all dysferlinopathy patients, demonstrating a high sensitivity and specificity for diagnosis of dysferlinopathy.

IH results, on the other hand, were misleading because dysferlin expression was deficient both in patients with mutations in *DYSF* and in patients with other myopathies. Most patients with *DYSF* mutations showed absence of dysferlin in IH, but some showed residual dysferlin expression. A large proportion of pathological controls with other myopathies (13/21) showed abnormal patterns of dysferlin expression using IH but their dysferlin expression in skeletal muscle and PBM was normal according to WB.

Several authors have described different IH patterns of dysferlin expression in muscle biopsies from patients with different myopathies, but none of them compared this expression with that found in PBM. The authors in the first of these studies found IH patterns which were slightly different from those in our study. However, they did not correlate their findings with muscle WB [15]. In two later papers, the authors observed mismatching results between WB and IH in muscle biopsies from different LGMD [14], [16]. In the paper by Tagawa *et al* the authors found defective expression of dysferlin by IH, 19% of patients with LGMD and 50% of patients with other neuromuscular diseases, and established four different patterns of dysferlin staining (normal, negative, faint and abnormal cytoplasmic accumulation) The authors concluded that other proteins are necessary for a normal localization of dysferlin at the sarcolemma. In the paper by Lo et al the authors found abnormal dysferlin expression in a significant proportion of LGMD patients not LGMD2B (25 out of 76). They grouped these patients in four categories according to IH pattern of dysferlin expression in the muscle biopsy including: 1) deficiency, 2) reduced membrane staining, 3) reduced membrane staining with homogenously increased sarcoplasmic staining in all fibers and 4) reduced membrane staining and variable sarcoplasmic staining in some fibers. To explain their results the authors suggested that either the membrane damage is too excessive for satellite cells to repair or that the proteins needed for dysferlin function are defective in these patients. The results of both studies matched well with those found in our study (Figure 4). In yet another study, the authors found a patchy pattern of sarcolemmal dysferlin in two patients with 5′ donor site mutations, but these mutations could not be related to a milder clinical phenotype [17].

In a more recent paper the authors described three patients with a suspicion of dysferlinopathy who displayed abnormal skeletal muscle IH (reduced or absent) and increased dysferlin protein by WB.In two of the three patients, genetic analysis showed a non-pathogenic change in one allele and no change in the second allele. The third patient displayed one pathogenic mutation in one allele and a non-pathogenic change in the second allele. WB in PBM was performed in two of the three patients and dysferlin expression was normal in both. The quantification method and the WB, however, were not shown [18]. Together with our findings these results indicate that normal dysferlin expression has not yet been observed when two pathogenic mutations in the *DYSF* gene are found.

Dysferlin interacts with other proteins to form a complex, such as annexin-1 and 2 [19], calpain-3 [20], [21], caveolin-3 [22], AHNAK [23], affixin [24], dihydropyridine receptor [25], MG53 [26] and tubulin [27]. Disruption of proteins in the dysferlin complex can affect the subcellular localization of dysferlin in the absence of mutations in the gene *DYSF* and may explain the abnormal immunohistochemical results described in the above-mentioned studies [14], [22], [28], [29], [30]. For this reason it is important to quantify dysferlin expression in skeletal muscle WB as well as by IH. However, in some cases the amount of muscle biopsy is insufficient to perform WB. It may also happen that in patients at end stages of the disease the muscle biopsy may mainly consist of fat and fibrotic tissue, also leading to inaccurate quantification results of muscle proteins. To overcome these difficulties, here we propose dysferlin expression should be studied in PBM to help interpret the reductions noted in the muscle biopsy by IH. Indeed, when the clinical suspicion of dysferlinopathy is high, dysferlin in PBM can be studied even before the muscle biopsy is taken. The mean expression of dysferlin by skeletal muscle WB in our patients was 1.1±4.3% and linked with that in monocytes. It is of note that the authors of a very recent report showed that when the expression of dysferlin in skeletal muscle analysed by WB was lower than 20% it was due to mutations in the gene *DYSF* [31]. The authors, however, did not compare dysferlin expression in PBM in the same patients, as we did in our study.

The analysis of dysferlin in PBM is very helpful to rule out a dysferlinopathy when no muscle sample is available. However, when available, the study of the muscle biopsy is very informative. In fact, the presence of characteristic inflammatory infiltrates [32] and sarcolemmal and interstitial amyloid deposits [33] can be helpful to support the diagnosis of dysferlinopathy and to study the pathogenesis of the disease.

The high parallelism between dysferlin expression in skeletal muscle and PBM by WB found in our study has improved the diagnostic approach in our routine practice, avoiding misleading diagnoses when the IH pattern is abnormal. In the diagnostic work up of a patient with proximal or distal weakness, we suggest to investigate the presence of dysferlin by WB in PBM in combination with the muscle biopsy prior to molecular analysis of a large gene such as *DYSF*.

## Materials and Methods

### Subjects

Patients were recruited at our neuromuscular unit, a reference center for dysferlin myopathy in Spain. The diagnosis of dysferlinopathy and other muscular dystrophies included the neurological examination and muscle MRI. Most cases also included a muscle biopsy to guide genetic analysis regarding the defective protein using IH and WB. PBM WB was carried out in 17 patients with two mutations in the *DYSF* gene and in 21 patients with other neuromuscular diseases. Our clinical research ethics committee approved this study (Reference number of our Ethics Committee: 08/77/883). Informed consent was obtained from all patients for muscle biopsy, blood samples and RNA/DNA analysis.

### Isolation of PBM and immunoblot analysis

PBM-CD14 positive isolation was carried out in all subjects as previously described [12]. WB from human monocyte samples was performed using a mouse monoclonal antibody to dysferlin (NCL-Hamlet, Novocastra, Newcastle, UK) and a mouse monoclonal antibody to β-actin (Sigma-Aldrich Quimica, Madrid, Spain) as a loading control. IRDye®800 conjugate goat anti-mouse was used as a secondary antibody (Li-cor, Lincoln, Nebraska, USA). Fluorescent bands corresponding to dysferlin and β-actin were acquired and analyzed using an Odyssey Infrared Imaging System (Li-cor). To determine dysferlin expression we quantified the double band observed in PBM. The control samples were used as reference values and assigned 100% of dysferlin expression using β-actin as a normalizer. The amount of dysferlin shows some variation among different healthy individuals. We used several healthy controls to perform the different dysferlin expression determinations, since the same individual control sample was not always available. When performing a WB we assign a value of 100% to a healthy individual that has no mutations in *DYSF*. For this reason, in a given gel, an individual with a pathology other that dysferlinopathy or in other healthy individuals the levels of dysferlin may be slighty higher or lower than 100%. Dysferlin expression in patient samples was quantified using the control values to obtain the percentages of dysferlin expression/reduction.

### Dysferlin knockdown

We performed an RNA inference experiment to confirm that the double band present in PBM corresponded to dysferlin. Human monocytes from healthy donors were isolated using the EasySep Human Monocyte Enrichment kit (Stemcell Technologies, Sheffield, UK) following the manufacturer's instructions.

230,000 monocytes/sample (100,000 monocytes/ml) were transfected with siRNA/siPORT Amine (Ambion, Texas, USA) at a concentration of 30 nM/5µl. siRNAs used were: siDYSF-1 target in exon 2 (s15788), siDYSF-2 target in exon 48 (s15790), siMYOF (s25476) as a non-related gene and a negative control (AM4611). Monocyte cultures were incubated with siRNAs for 6 days and dysferlin protein was analyzed by WB.

### Immunoprecipitation

Freshly isolated PBM were lysed on ice in buffer composed of 50 mM Tris-HCl pH 7.5, 150 mM NaCl, 0.15% CHAPS and 1× protease inhibitor cocktail. The lysates were spun down at maximum speed, 4°C, 20 min. Protein A Sepharose CL-4B (Amersham) was washed 3× in lysis buffer and used to preclear the homogenates for 1 h, at 4°C while shaking. Sepharose was removed and antibody was added (50 µg Heavy Chain Antibody (HCAb) fragment) and shaken o/n at 4°C. HCAb fragments F4 (amino acids 2–245) and H7 (amino acids 1666–1788) (both Dysferlin specific) (Figure 2A and 3A) (non-specific control against amyloid-β 34) were described previously [21], [34]. The washed Sepharose was then added, incubated while shaking for 2 h at 4°C. Bound and non-bound IP samples were separated on SDS-page gels and blotted onto a nitrocellulose membrane. Primary antibody (Hamlet) against amino acids 1999–2016, was incubated o/n and secondary antibody for 1 h. Detections were performed with an Odyssey scanner (Li-Cor).

### Mutation analysis of *DYSF*

Mutation screening in the *DYSF* gene was performed in 25 patients, the seventeen with abnormal dysferlin by WB and eight patients from the pathological control group with normal levels of dysferlin protein in WB from muscle and monocytes and no final diagnosis of their myopathy. The RNA was extracted from PBM using Ultraspec (Biotech Laboratories, Houston, USA) and retrotranscribed with the oligo(dT) primer technique (Invitrogen, Carsbad, USA). Amplification and direct sequencing of the entire dysferlin cDNA was performed using 14 sets of oligonuclotide primers covering the 55 exons, as previously described [35].

### Skeletal muscle immunohistochemistry and immunoblot

We studied dysferlin expression in muscle in 38 biopsy samples. Immunohistochemistry was performed as previously described [36]. The slides were incubated overnight with a primary antibody to human dysferlin (NCL-Hamlet) at a final concentration of 10 µg/ml. After a washing step, slides were incubated with a secondary antibody for 1 hour at room temperature. Samples were then mounted and directly examined using an Axioscop 2 plus photomicroscope equipped with epifluorescence optics and bright field (Carl Zeiss, Munich, Germany). Lysates from the same 38 skeletal muscle biopsies were analyzed by WB as previously described [3], [37]. Briefly, frozen muscle samples were homogenized with 19 volumes of treatment buffer containing 0.125 mol/L Tris/HCL buffer pH 6.4, 10% glycerol, 4% SDS, 4 mol/L urea, 10% mercaptoethanol and 0.001% bromophenol blue and 30 µl of samples were loaded in the gel (equivalent to 200 µg of non-collagen protein). Immunoblots were performed as described above for monocytes.

## Supporting Information

Figure S1 Western blot of skeletal muscle and PBMs from 2 patients and 2 controls all loaded on the same gel using an antibody to dysferlin (Hamlet).
(TIF)
