# Supplementary figures and images for: Comparison of Dysferlin Expression in Human Skeletal Muscle with That in Monocytes for the Diagnosis of Dysferlin Myopathy
Source: PLoS One. 2011 Dec 16;6(12):e29061. doi: 10.1371/journal.pone.0029061 (PMC3241698; doi:10.1371/journal.pone.0029061)

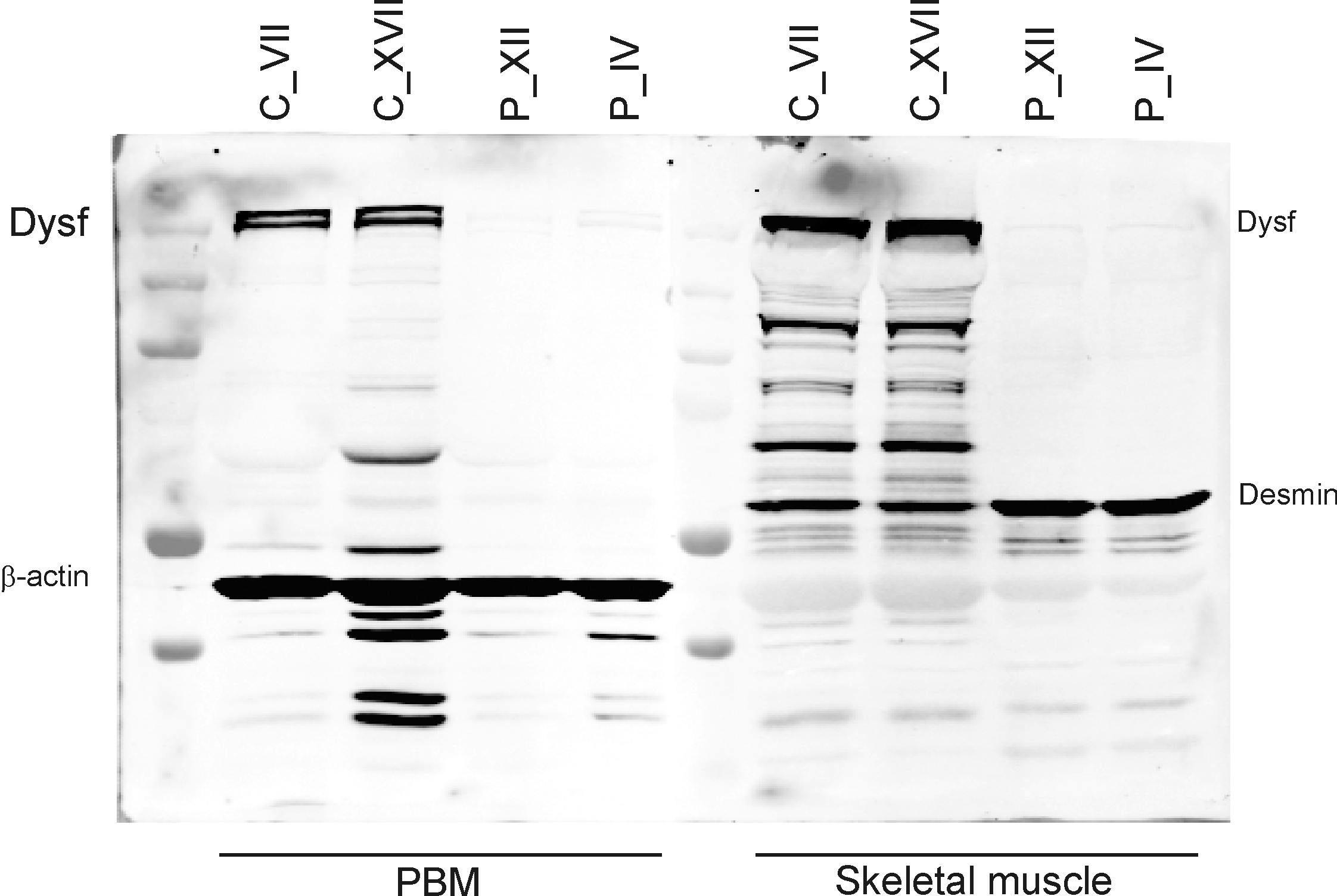

Supplement: Figure S1 — Western blot of skeletal muscle and PBMs from 2 patients and 2 controls all loaded on the same gel using an antibody to dysferlin (Hamlet). (TIF) [file pone.0029061.s001.tif]
